# Primary cilia regulate gastric cancer-induced bone loss via cilia/Wnt/β-catenin signaling pathway

**DOI:** 10.18632/aging.202734

**Published:** 2021-03-09

**Authors:** Jie Xu, Xiaoyan Deng, Xiangmei Wu, Huifang Zhu, Yinghua Zhu, Jie Liu, Qian Chen, Chengfu Yuan, Geli Liu, Changdong Wang

**Affiliations:** 1 Department of Biochemistry and Molecular Biology, Molecular Medicine and Cancer Research Center, College of Basic Medicine, Chongqing Medical University, Chongqing 400016, China; 2 Department of Physiology, Molecular Medicine and Cancer Research Center, College of Basic Medicine, Chongqing Medical University, Chongqing 400016, China; 3 Department of Pre-Hospital Emergency, Chongqing Emergency Medical Center, Central Hospital of Chongqing University, Chongqing 400014, China; 4 College of Medical Science, China Three Gorges University, Yichang 443002, Hubei, China

**Keywords:** gastric cancer, bone loss, cilia, Wnt/β-catenin signaling pathway

## Abstract

Cancer-associated bone disease is a frequent occurrence in cancer patients and is associated with pain, bone fragility, loss, and fractures. However, whether primary or non-bone metastatic gastric cancer induces bone loss remains unclear. Here, we collected clinical evidence of bone loss by analyzing serum and X-rays of 25 non-bone metastatic gastric cancer patients. In addition, C57BL mice were injected with the human gastric cancer cell line HGC27 and its effect on bone mass was analyzed by Micro-CT, immunoblotting, and immunohistochemistry. Furthermore, the degree of the proliferation and osteogenic differentiation of mesenchymal stem cells (MSCs) co-cultured with HGC-27 or SGC-7901 cells was analyzed by colony-formation assay, alizarin red staining, immunofluorescence, qPCR, immunoblotting, and alkaline phosphatase activity assay. These indicated that gastric cancer could damage bone tissue before the occurrence of bone metastases. We also found that cilia formation of MSCs was increased in the presence of HGC27 cells, which was associated with abnormal activation of the Wnt/β-catenin pathway. Expression of DKK1 inhibited the Wnt/β-catenin signaling pathway and partially rescued osteogenic differentiation of MSCs. In summary, our results suggest that gastric cancer cells might cause bone damage prior to the occurrence of bone metastasis via cilia-dependent activation of the Wnt/β-catenin signaling pathway.

## INTRODUCTION

While bone loss is likely to occur during the early stages of gastric cancer, it has largely remained undetected and has therefore not caused much concern. The occurrence of cancer-associated bone disease may be due to the direct or systemic action of the gastric cancer itself during the treatment of the primary tumor. Bone loss is associated with localized effects of metastatic deposits in the bone and/or systemic bone loss caused by bone resorption hormones or cytokines released into the systemic circulation by the gastric cancer [1]. Frequently, mesenchymal and hematopoietic progenitors are associated with the action of parathyroid hormone-related protein (PTHrP), a well-known regulator of tumour-associated bone destruction [1], as well as with the hypercalcemia seen in many types of cancer [2]. Once bone metastases occur, gastric cancer-induced osteoclast activation and bone resorption can proceed at a dangerously high rate resulting in the development of bone lesions that cause considerable pain and morbidity in patients [3]. However, the phenomenon of bone loss caused by gastric cancer has not received enough clinical attention, and its molecular mechanism and optimal treatment strategy are still unknown. In this study we focus on bone loss during the early stages of gastric cancer development before the occurrence of bone metastasis.

Low bone mass and strength lead to fragility and fractures, for example in elderly individuals affected by osteoporosis or children suffering from osteogenesis imperfecta [4]. A decade ago, rare human mutations were identified that either negatively (osteoporosis pseudoglioma syndrome) or positively (high-bone mass phenotype, sclerosteosis and Van Buchem disease) affect bone formation. All of these mutations were associated with components of the canonical Wnt signaling machinery [4].

Historically, wingless-INT (Wnt) signaling has been subdivided into three major branches: the Wnt/β-catenin pathway, also termed the canonical Wnt pathway [5], the noncanonical Wnt/planar cell polarity [6] pathway and the Wnt/calcium (Wnt/Ca^2+^) pathway [7]. The fact that the canonical pathway emerged as the predominant component of Wnt signaling affecting bone cells has allowed the field to put together a relatively clear picture of the mechanisms by which Wnt affects the skeleton [5]. Wnt signaling pathways exert distinct effects during different phases of bone development, including chondrogenesis, osteoblastogenesis, and osteoclastogenesis [8–12]. Wnt/β-catenin signaling is initiated upon binding of canonical Wnt ligands to a dual receptor complex comprising the Wnt co-receptors LRP5 or LRP6 (LRP5/6) and one of the seven transmembrane receptors of the FZD family [5]. Axin is located to the LRP5/6 tail at the membrane through its interaction with dishevelled (DVL, also called DSH), which is recruited by FZD [5]. This forms a complex that also includes FRAT1 and GSK3β, which prevents phosphorylation of β-catenin and its proteosomal degradation [5]. Then, β-catenin accumulates in the cytoplasm and translocates into the nucleus, where it associates with members of the TCF/LEF transcription factors while displacing Groucho to control target gene transcription [5].

Primary cilia are found in almost every mammalian cell type [13], and act as the "antennae" of cells that can sense and transduce signals from the microenvironment, especially through Wnt signaling [14], and have emerged as a major regulator of Wnt signaling [15]. Intraflagellar transport 80 (Ift80) regulates the growth of fibril hairs and is responsible for transmitting signaling substances from the bottom to the top of the cilia. In recent years, skeletal abnormalities have been found in human cilia-related diseases [16–19] and IFT-related mouse gene knockout studies [20–22] have indicated a functional importance of cilia for bone development. A number of studies have shown that primary cilia regulate the skeletal development of adult embryos [20, 23–26] and mechanically regulate the formation of adult bones [27, 28]. The important role of cilia in skeletal development and formation is well documented [27, 28], but it was not until recently that the role of cilia in cancer development has begun to receive attention. It has been reported that in the case of pancreatitis [29], cilia are sharply reduced during the formation of pancreatic ductal adenocarcinoma [30]. Similarly, in prostate cancer loss of cilia is associated with increased Wnt/β-catenin activity and increased malignant characteristics. Importantly, some cancers depend on retaining rather than losing cilia. There are different fibrotic patterns in fibroblastoma and basal cell carcinoma that affect their ability to respond to additional cellular signals with ciliary receptors [31, 32]. However, the function and mechanism of cilia in gastric cancer-induced bone loss is unclear.

In this study, we collected 25 clinical specimens of early gastric cancer patients and non-bone metastatic gastric cancer patients. We found that some of these 25 patients had low serum calcium and phosphorus concentrations, unstable alkaline phosphate (ALP) concentrations, and apparent bone loss in computed tomography (CT) films. Using *in vitro* and *in vivo* experiments we demonstrated that the Wnt/β-catenin signaling pathway was the main pathway for gastric cancer-induced bone loss. Our results showed that gastric cancer cells stimulated cilia formation in bone cells leading to nuclear translocation of β-catenin, which further activated the Wnt signaling pathway. As a result, gastric cancer ultimately affected the degree of osteogenic differentiation, resulting in impaired bone formation and eventually in the occurrence of bone loss. In agreement with these results, DKK1, a specific Wnt/β-catenin inhibitor, significantly promoted osteoblast differentiation and induced bone formation.

## RESULTS

### Clinical samples reveal that primary gastric cancer induces bone loss prior to the occurrence of bone metastasis

**Figure 1:**
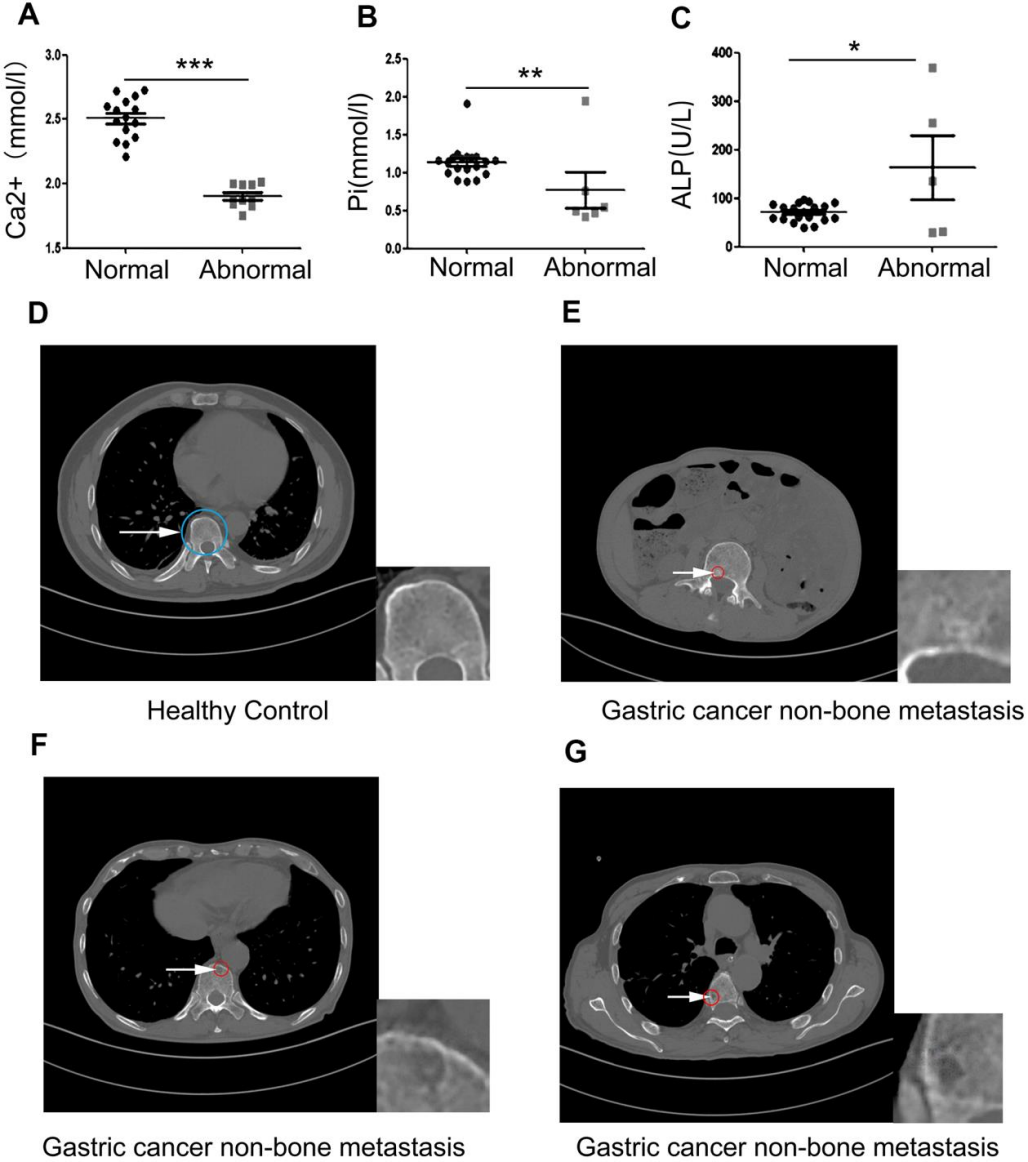
**Clinical samples reveal that primary gastric cancer induces bone loss before bone metastasis occurs.** (**A**–**C**) The concentration of serum Ca^2+^, Pi, and ALP in 25 non-bone metastatic gastric cancer patient compared to healthy volunteers. (**D**) Healthy control’s pyramidal computed tomography (CT). (**E**–**G**) Bone sections of three individual patients with non-bone metastatic gastric cancer. The insets are showing a high-power image of bone loss. The blue circle indicates normal bone and the white arrow pointing to the red circle indicates damaged bone. Data are shown as mean±SEM. Statistical differences are obtained using a Student's t-test, *, p<0.05, **, p<0.01, ***, p<0.001.

The concentration of Ca^2+^, inorganic phosphate (Pi), and ALP in the serum of 25 gastric cancer patients with non-bone metastases who met the study inclusion criteria, and representative CT films of bone loss were collected. Data showed that 11 of 25 patients had an abnormal serum Ca^2+^ index, 6 patients had an abnormal serum Pi index and 5 patients had an abnormal ALP index. Serum levels of Ca^2+^ and Pi rapidly decreased (Figure 1A, 1B) compared with the normal value. However, levels of ALP had dramatically increased compared with the normal value (Figure 1C). ALP is a membrane-bound enzyme required to provide the cells with the necessary source of inorganic Pi by hydrolyzing pyrophosphate. Since humans are made up of complex systems, this suggested that the increase in ALP was caused by the destruction of bone. These data demonstrated that the bones of these gastric cancer patients were damaged before the occurrence of bone metastases. Next, we set out to obtain the CT myelograms of our cohort of gastric cancer patients. Since physicians typically pay little attention to the possible impact of early gastric cancer on bone health, it was not possible to obtain myelographies from all 25 patients. However, we managed to obtain myelographies from 3 patients and from these we could deduce that distinct bone loss had occurred. Compared with healthy controls, a slight circular high-density shadow was visible in the vertebral body in the first myelography (white arrow pointing to the red circle in Figure 1E); a small area of low-density shadow was seen in the second myelography, but the shadow density was slightly higher (Figure 1F); In the third myelography, a small, low-density shadow was visible in the vertebral body (Figure 1G). These results confirmed that gastric cancer induced bone loss before bone metastasis had occurred.

### C57BL mice injected with HGC27 *in vivo* confirms that gastric cancer induces bone loss

**Figure 2:**
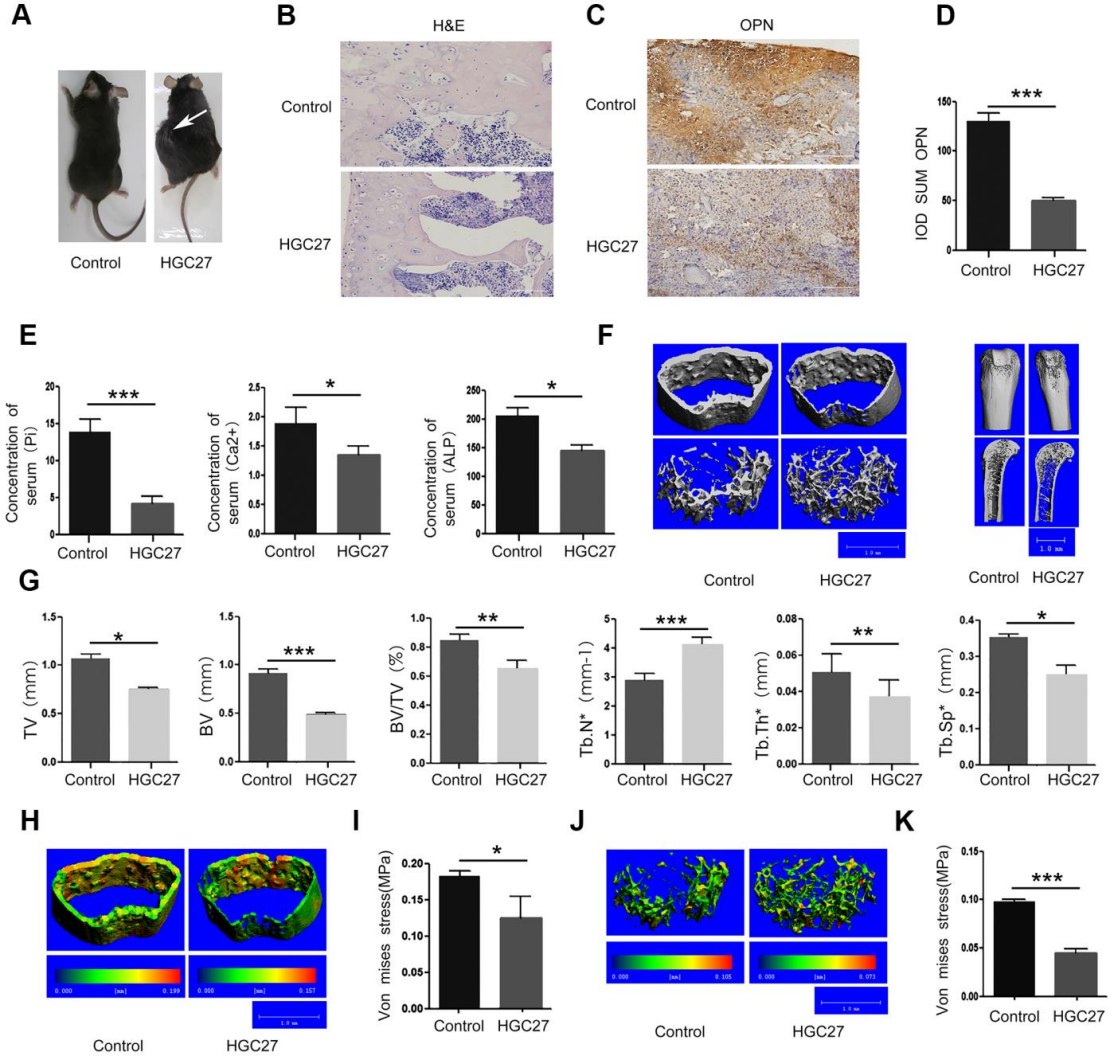
**Injection of C57BL mice with HGC27 cells *in vivo* confirms that gastric cancer induces bone loss.** (**A**) C57BL mice were injected with HGC27 cells or PBS (control). Mice were observed for 90 days following the injection. The white arrow indicates the localization of the gastric tumor. (**B**) H&E staining of isolated femurs from the control group and the experimental group. Scale bar, 100μm. (**C**) Immunohistochemical staining of osteopontin (OPN) from representative normal and damaged femoral tissues. The brown color indicates OPN-positive cells. Scale bar, 100 μm. (**D**) Comparison of IOD SUM of OPN-positive cells in (**C**). (**E**) Peripheral blood of C57BL mice injected with PBS or HGC27 cells was collected 90 days post-injection for determination of serum phosphorus (Pi), calcium (Ca^2+^) and alkaline phosphatase (ALP) respectively. (**F**) Micro-CT showing the transverse section and longitudinal section of the femur. (**G**) Quantitative analysis of the percentage of total bone mass (TV), bone mass (BV), bone volume to total bone volume (BV/TV), trabecular number (Tb.N*), trabecular thickness (Tb.Th*) and trabecular spacing (Tb.Sp*) in femurs from the two groups at day 90. (**H**) Micro-CT showing a comparison of the stress levels of the femoral cortical bone. (**I**) Quantitative analysis of the ability of the femoral cortex to withstand stress. (**J**) Micro-CT showing a comparison of the stress tolerance of the femoral trabecular cancellous bone. (**K**) Quantitative analysis of the femoral trabecular cancellous bone portion subjected to stress. Data are shown as mean±SEM. Statistical differences were obtained using a Student's t-test, *, p<0.05, **, p<0.01, ***, p<0.001. n=3 per-group.

To investigate whether non-bone metastasis of gastric cancer may induce bone loss, we investigated the effect of HGC27 cells on *in vivo* tumor growth using a mouse xenograft model. We subcutaneously injected 2x10^9^ HGC27 cells into the flank of C57BL mice and monitored tumor growth for 90 days. In Figure 2A, the white arrow points to gastric cancer tissue in mice that were injected with HGC27 while no tumors developed in the control mice that were injected with phosphate-buffered saline (PBS). Bone tissues from both groups were dissected for histological H&E staining. We found that the HGC27-injected group showed obvious vacuoles and osteoporosis, but no signs of bone metastasis (Figure 2B and Supplementary Figure 1). Next, we tested the mice for the presence of the osteoblast differentiation marker osteopontin (OPN), and found a significant decrease in OPN-positive areas in the HGC27-injected group compared with control group (Figure 2C). Mean optical density analysis confirmed that the OPN-positive region was significantly reduced in the HGC27-injected group compared with the control group (Figure 2D). Peripheral blood sample measurements showed a significant decrease in Pi, Ca^2+^, and ALP concentrations in the HGC27-injected group compared with the control group (Figure 2E). Micro-computed tomography (Micro-CT) of femurs of HGC27-injected C57BL mice at day 90 showed an apparent bone loss in both trabecular and cortical bone (Figure 2F) compared with controls. Bone volume (BV), total bone mass (TV), ratio of bone volume to total bone mass (BV/TV), trabecular thickness (Tb.Th*), and trabecular number (Tb.Sp*) in HGC27 injected mice were 0.54, 0.70, 0.77, 0.71, 0.71 fold, respectively, of that of the control. Although the trabecular number (Tb.N*) of the HGC27-treated group was almost 1.44 fold of that of the control group (Figure 2G), the HGC27 group had significantly lower bone mineral density and a 30% reduction in osteophyte tolerance (Figure 2H–2J). Meanwhile, cancellous bone resistance was reduced by 60% compared to the control group (Figure 2I–2K). These *in vivo* results indicated that injection of HGC27 induced bone loss and significantly reduced bone stress tolerance, consistent with our clinical results.

### Osteoblasts co-cultured with HGC27 or SGC-7901 *in vitro* confirm that gastric cancer inhibit the proliferation and osteoblast differentiation

**Figure 3:**
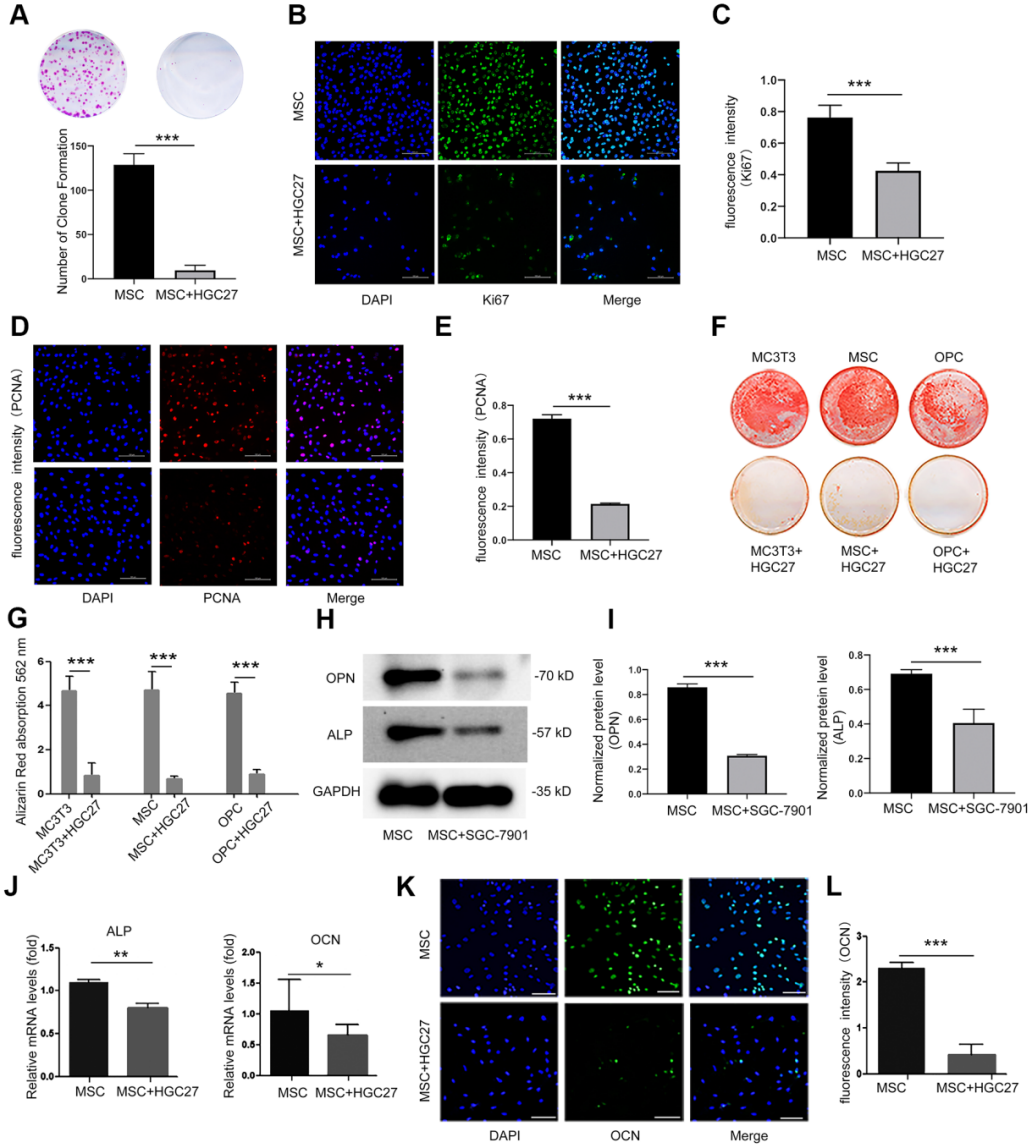
**Co-culturing of osteoblasts with HGC27 or SGC-7901 cells *in vitro* confirms that gastric cancer cells inhibit the proliferation and differentiation of osteoblasts.** (**A**) Colony formation assay showing that the proliferation ability of MSC cells was impaired upon co-culture with HGC27 cells. (**B**) Immunofluorescence staining of MSCs at day 3 of OS medium induction with or without co-cultured HGC27. Shown is Ki67 expression (green). Nuclei were stained with DAPI (blue). Scale bar, 100 μm. (**C**) Quantitative analysis of the fluorescence intensity in (**B**). (**D**) Immunofluorescence staining of PCNA (red) in MSCs at day 3 of OS induction with or without co-cultured HGC27 and Nuclei were stained with DAPI. (blue) states. Scale bar, 100 μm. (**E**) Quantitative analysis of the fluorescence intensity in (**D**). (**F**) Alizarin Red staining analysis of MC3T3, MSC, and OPC cells at days 16 of OS induction with or without HGC27 cells co-cultured. (**G**) Quantitative mineralization level based on (**F**). (**H**) Western blot analysis of OPN and ALP expression in MSCs with or without co-cultured SGC-7901. (**I**) Quantitative analysis of OPN and ALP protein levels from the immunoblots in (**H**). OPN and ALP protein levels were normalized to GAPDH (n=3). (**J**) qRT-PCR results showing ALP and OCN genes transcription levels in MSCs at day 3 of OS induction with or without co-culturing of HGC27. Expression levels of ALP and OCN were normalized to GAPDH expression. (**K**) Immunofluorescence staining of MSCs at day 3 of OS induction with or without co-cultured HGC27 to test OCN (green). Nuclei were stained with DAPI (blue) states. Scale bar, 100 μm. (**L**) Quantitative analysis of the fluorescence intensity in (**K**). Data are shown as mean±SEM. Statistical differences were obtained using Student's t-test, *, p<0.05, **, p<0.01, ***, p<0.001. n=3 per-group. OPC, oligodendrocyte progenitor cells; OPN, osteopontin; ALP, alkaline phosphatase; MSC, mesenchymal stem cells.

To further determine the existence of gastric cancer-induced bone loss, we used a colony formation assay to analyze the effect of HGC27 cells on the proliferation of MSCs. Our results showed that the presence of HGC27 caused the inhibition of the proliferation of MSCs (Figure 3A). Assessment of Ki67 and PCNA confirmed that the proliferation ability of MSCs co-cultured with HGC27 had decreased compared with the control group (Figure 3B–3E). Moreover, three kinds of osteoblast cells (MC3T3, MSC, and OPC) were grown in the presence of HGC27 cells for transwell co-culture experiments. Subsequent alizarin red staining indicated that the differentiation of osteoblasts was significantly reduced upon co-culture with HGC27 compared with controls, with an almost complete absence of calcium ion deposition (Figure 3F). After quantitative analysis, the calcium nodules in the experimental groups were reduced by 82% (MC3T3), 61% (MSC), and 81% (OPC), respectively, compared with the control group (Figure 3G). The use of MSCs in co-culture models allowed us to study the process of osteogenesis. Western blotting revealed that the expression of marker proteins of osteogenic differentiation, including ALP and OPN, in MSCs co-cultured with SGC-7901 was significantly lower than in the control (P<0.001; Figure 3H, 3I). Therefore, we used MSCs as cell model for co-culture in the following experiments. RNA was extracted for qRT-PCR analysis at day 3 with HGC27 co-cultured. The levels of osteogenic marker genes ALP and OCN had both significantly decreased compared to control group (P<0.01 and P<0.5, respectively; Figure 3J). In addition, we performed immunofluorescence experiments at day 3 of the HGC27 co-culture experiments and found that the protein expression of OCN was significantly reduced compared to control (Figure 3K, 3L). These data were consistent with the bone loss observed in the *in vivo* model, highlighting that gastric cancer reduced osteoblast differentiation.

### Co-culture with HGC27 cells but not GES-1 cells increased cilia formation of MSCs

**Figure 4:**
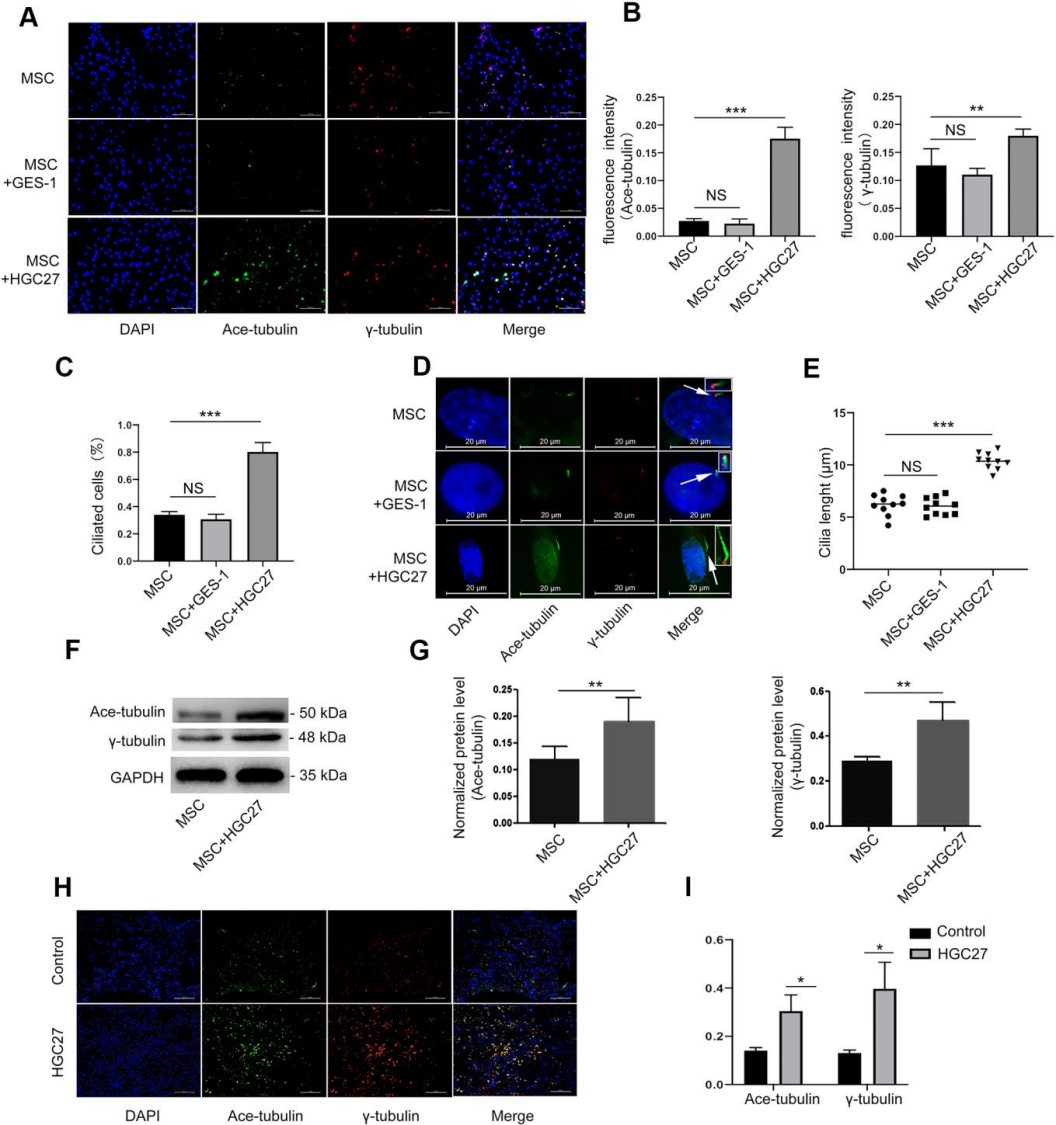
**HGC27 but not the GES-1 increased the cilia formation of MSCs.** (**A**) Immunofluorescence analysis of primary cilia in MSC, MSC+GES-1, MSC+HGC27. Primary cilia were stained with acetylated α-tubulin (axoneme, green) and γ-tubulin (basal body, red) antibody. Scale bars, 100 μm. (**B**) Quantitative analysis of the fluorescence intensity of immunofluorescence in (**A**). (**C**) Quantitative evaluated the number of cilia on an equal area (n=3, at least 200 cells). (**D**) Representative images of primary cilia in MSCs. The inset shows a high-power image of the basal body and axoneme. Primary cilia are stained with acetylated α-tubulin (axoneme, green) and γ-tubulin (basal body, red) antibodies. DAPI staining is used as nuclear counterstaining (blue). Scale bars, 20 μm. (**E**) Graphical representation of cilia length in the MSC, MSC+GES-1, and MSC+HGC27 groups (n=10). (**F**) Western blot analysis of acetylated α-tubulin and γ-tubulin expression in MSCs with or without co-cultured HGC27. (**G**) Quantitative analysis of acetylated α-tubulin and γ-tubulin protein levels from immunoblots in (**F**). Acetylated α-tubulin and γ-tubulin protein levels were normalized to GAPDH (n=3). (**H**) Femur tissues of C57BL mice injected with PBS or HGC27 cells at 90 days post-injection are stained for γ-tubulin (red), acetylated α-tubulin (green), and nuclei (blue). Scale bar, 100 μm. (**I**) Quantitative analysis of the fluorescence intensity in (**H**). Data are shown as mean±SEM. Statistical differences were obtained using Student's t-test, *, p<0.05, **, p<0.01, ***p<0.001.

Then, we set out to assess whether gastric cancer cells could affect osteoblastic cilia. We performed cellular immunofluorescence assays on MSCs, MSCs co-cultured with GES-1 cells, and MSCs co-cultured with HGC27 cells and double stained basal bodies (using anti-γ-tubulin) and axons (using anti-acetylated α-tubulin) to visualize cilia (Figure 4A). MSC co-cultured with GES-1 cells displayed almost the same cilia expression as control MSCs. In contrast, MSC co-cultured with HGC27 cells showed abnormally increased cilia expression (Figure 4A, 4B). About 35% of the cells in the control MSC group had normal cilia structure, whereas the number of cilia in the MSCs co-cultured with HGC27 cells was as high as 78% (Figure 4C). Moreover, staining with anti-acetylated α-tubulin revealed a significant increase in the cilia length of MSCs co-cultured with HGC27 cells (10.15±1.35μm) compared to control MSCs (5.98±0.5μm) (Figure 4D and 4E). In addition, some MSCs also had 3-5 cilia. Staining with anti-γ-tubulin antibodies revealed a number of red dots that were distributed on the endoplasmic reticulum or the nucleus of the cell (Figure 6A). Western blot results confirmed that the expression of acetylated α-tubulin and γ-tubulin proteins was higher in MSCs co-cultured with HGC27 cells than in the control group (Figure 4F, 4G). These results were verified by subsequent animal experiments injecting mice with HGC27 cells. Bone tissues from the control mice and from HGC27-injected mice were subjected to immunofluorescence staining. The basal body (anti-γ-tubulin) and axoneme (anti-acetylated α-tubulin) were double-stained to detect cilia (Figure 4H). Quantitative analysis of immunofluorescence results using image J software showed that the expression levels of γ-tubulin and acetylated α-tubulin increased significantly in the HGC27-injected group compared with the control group (Figure 4I). In summary, HGC27 cells but not GES-1 cells promote the formation of cilia.

### Gastric cancer cells activate the canonical Wnt/β-catenin signaling pathway in bone

**Figure 5:**
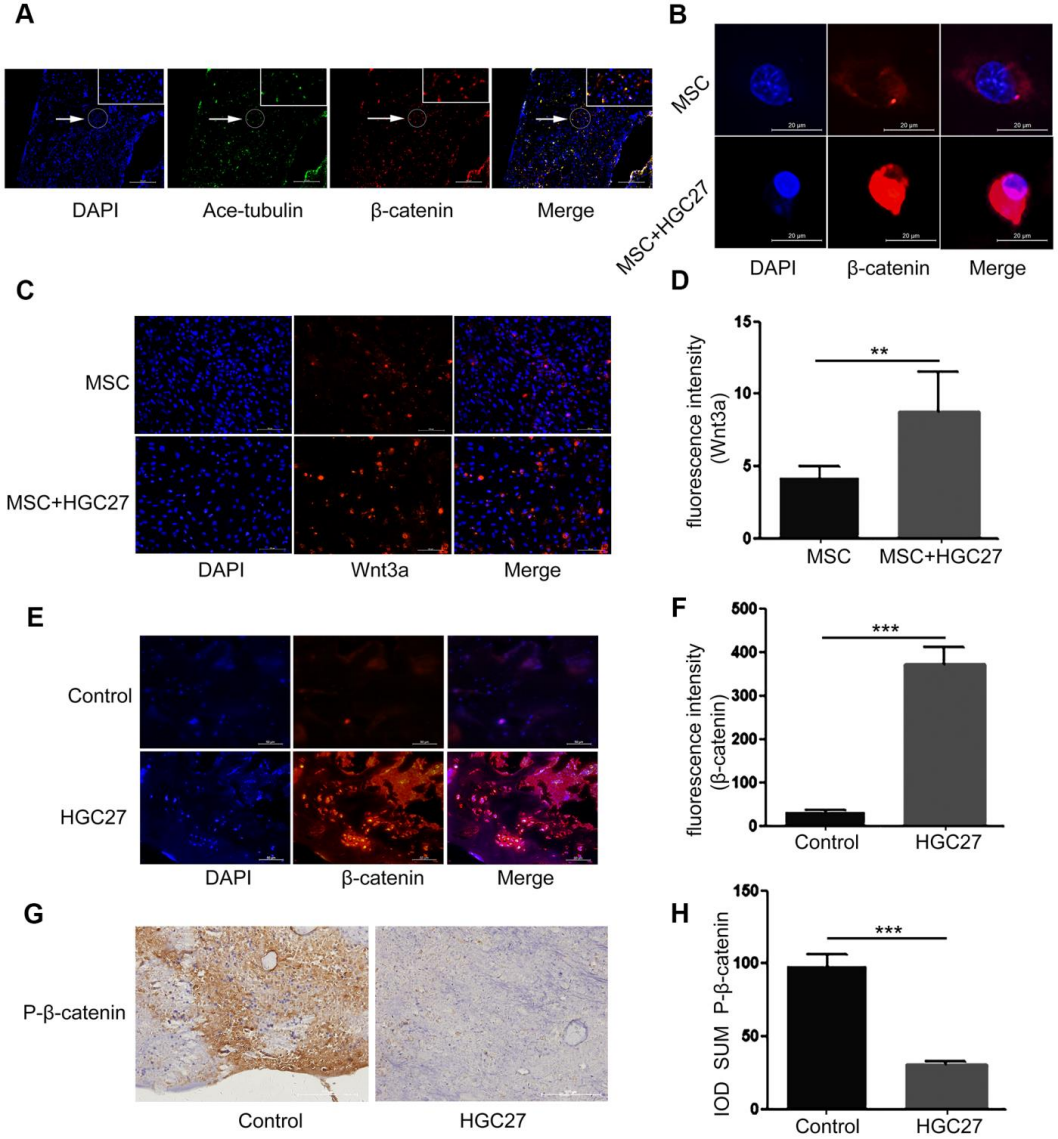
**Gastric cancer activates the canonical Wnt/β-catenin signaling pathway in bone.** (**A**) Immunofluorescence analysis of acetylated α-tubulin (ciliary axonin, green) and β-catenin (red) in femur from PBS injected C57BL mice. DAPI (nuclear marker, blue) staining was used as counterstain. Scale bars, 100 μm. (**B**) Immunofluorescence staining of MSCs with or without co-cultured HGC27 to visualize β-catenin (red) and nuclei (DAPI, blue) states. Scale bar, 20 μm. (**C**) Immunofluorescence staining of MSCs with or without co-cultured HGC27 showing Wnt3a (red) and nuclei (blue). Scale bar, 100 μm. (**D**) Quantification of immunofluorescence intensity in (**C**). (**E**) Representative femoral tissues from C57BL mice injected with PBS or HGC27 were analyzed for β-catenin expression by immunofluorescence staining at day 90. Scale bar, 50 μm. (**F**) Quantification of immunofluorescence intensity in (**E**). (**G**) Immunohistochemical staining for phosphorylated-β-catenin (p-β-catenin) in femurs of PBS or HGC27-injected groups. Scale bar, 100 μm. (**H**) IOD SUM of positive cells from (**G**) were compared between the HGC27 group and control femurs. Data are shown as mean±SEM. Statistical differences were obtained using Student's t-test, **, p<0.01, ***, p<0.001. n=3 per-group.

It is well known that the Wnt/β-catenin pathway regulates the production of cartilage, osteogenic cells and osteoclasts. It plays a key role in limb formation and bone development. Here we explored whether the Wnt/β-catenin signaling pathway also plays a role in gastric cancer-induced bone loss. We therefore performed immunofluorescence co-staining assays for β-catenin and acetylated α-tubulin (ciliary axon protein) on bone tissue of normal C57BL mice. Surprisingly, the expression sites of these two proteins overlapped highly (Figure 5A). These data demonstrated that β-catenin is already strongly associated with cilia under normal conditions. Next, we used immunofluorescence staining to determine the expression of β-catenin in MSCs that were co-cultured with or without HGC27 at day 3 of osteogenic (OS) medium induction. The results indicated (Figure 5B) that β-catenin (shown in red) expression was low under normal conditions and mostly distributed in the cytoplasm. However, accumulation of β-catenin in the cytoplasm and progressive translocation to the nucleus was observed at day 3 in MSCs co-cultured with HGC27 (Figure 5B). Similarly, we used immunoblotting to detect Wnt3a protein. The results showed that the expression of Wnt3a was 2.11-fold higher in MSCs co-cultured with HGC27 than in control MSCs, suggesting the abnormal activation of Wnt3a expression in MSCs by gastric cancer cells (Figure 5C, 5D). *In vivo* we collected femurs at day 90 from C57BL mice injected with HGC27 or PBS as control for β-catenin staining. Similar to the in *vitro* results, β-catenin expression was significantly increased in the HGC27 group, i.e., 12.1-fold higher than in the control group (Figure 5E, 5F). Meanwhile, immunohistochemical assays showed that the protein expression level of P-β-catenin (inactive state) in the HGC27 group was reduced by 84% compared with the control group (Figure 5G, 5H). Taken together, these data suggested that gastric cancer cells activate the expression of Wnt/β-catenin pathway during the process of osteoblast differentiation in bone.

### Abnormal overexpression of cilia caused by gastric cancer elevates the Wnt/β-catenin signaling pathway in MSCs

**Figure 6:**
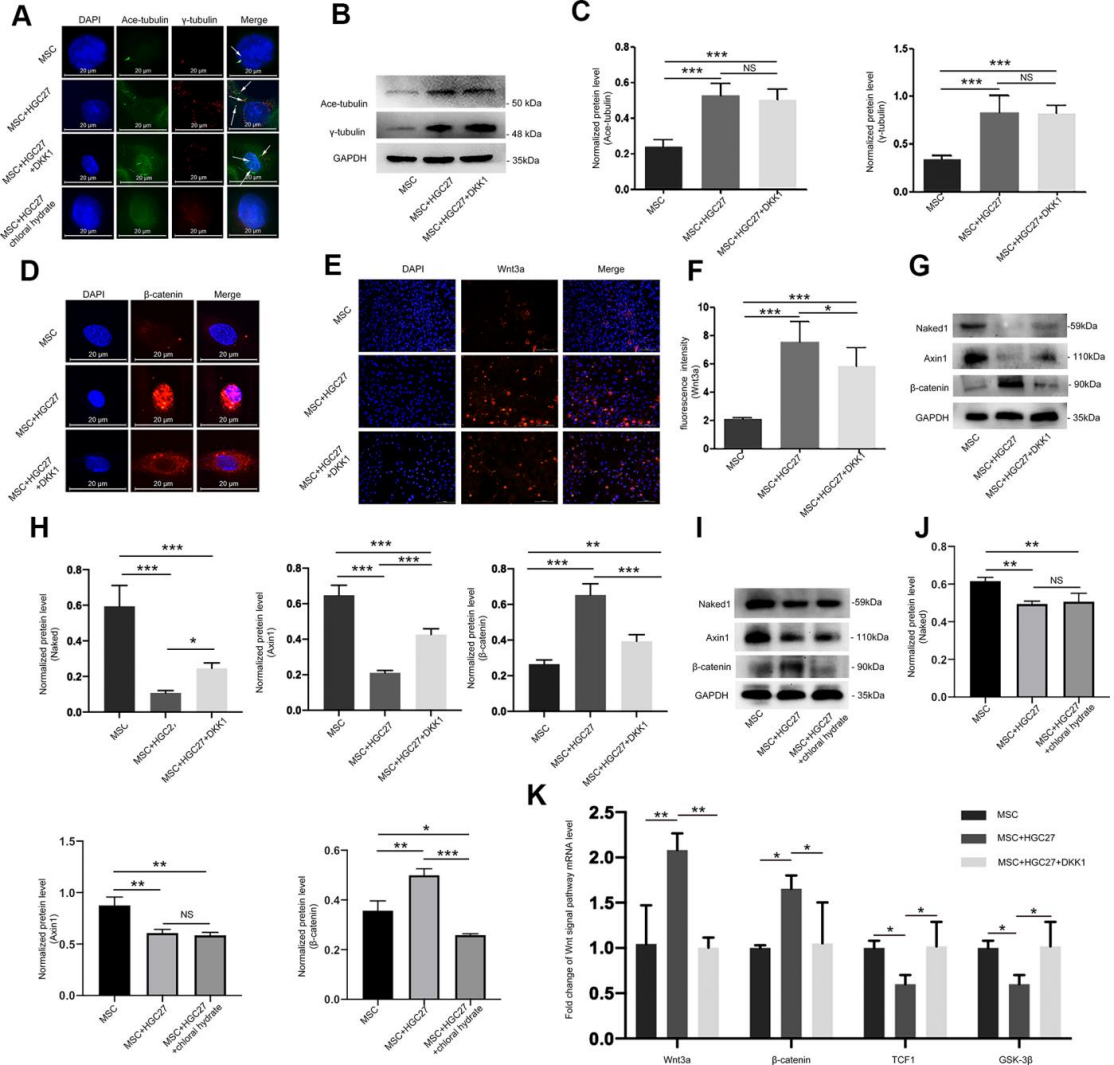
**Abnormal overexpression of cilia caused by gastric cancer activates the Wnt/β-catenin signaling pathway in MSCs.** (**A**) Analysis of primary cilia in MSC, MSC+HGC27, MSC+ HGC27+DKK1 and MSC+HGC27+chloral hydrate by immunofluorescence. Primary cilia were stained with anti-acetylated α-tubulin (axoneme, green) and anti-γ-tubulin (basal body, red) antibodies. Nuclei were stained with DAPI (blue). White arrows indicate cilia. Scale bars, 20 μm. (**B**) Western blot analysis of acetylated α-tubulin and γ-tubulin expression in MSC, MSC+HGC27 and MSC+HGC27+DKK1. (**C**) Quantitative analysis of protein levels in (**B**). Protein levels were normalized to GAPDH. (**D**) Analysis of β-catenin (red) expression by immunofluorescence in MSC, MSC+HGC27, and MSC+HGC27+DKK1 groups. Nuclei were stained with DAPI (blue). Scale bars, 20 μm. (**E**) MSC, or MSC co-cultured with HGC27 with or without DKK1 treatment were analyzed for Wnt3a (red) expression. Nuclei were stained with DAPI (blue). Scale bar, 100 μm. (**F**) Quantitative analysis of the fluorescence intensity in (**E**). (**G**) Western blot analysis comparing Naked1, Axin1, or β-catenin expression in the MSC, MSC+HGC27, and MSC+HGC27+DKK1 groups. (**H**) Quantitative analysis of protein levels in (**G**). The protein levels were normalized to GAPDH. (**I**) Western blot analysis comparing Naked1, Axin1, or β-catenin expression in the MSC, MSC+HGC27 and MSC+HGC27+chloral hydrate groups. (**J**) Quantitative analysis of protein levels in (**I**). The protein levels were normalized to GAPDH. (**K**) qRT-PCR results showing Wnt3a, β-catenin, TCF-1, and GSK-3β transcription levels in MSC, MSC+HGC27, MSC+HGC27+DKK1 on day 3 following OS induction. The gene expression levels were normalized to GAPDH expression. Data are shown as mean ± SEM. Statistical differences were obtained using One-way ANOVA with post-hoc testing, *, p<0.05, **, p<0.01, ***, p<0.001. NS, not statistically significant, n=3, per-group. Ace-tubulin, acetylated α-tubulin.

Our previous experiments had shown that gastric cancer up-regulated cilia expression in osteoblasts. Here, we further set out to characterize the relationship between cilia and Wnt/β-catenin pathway in gastric cancer leading to bone loss. Chloral hydrate causes degradation of the junction of the cilia and the matrix, and chemically blocks the formation of cilia. Dkk1 is a classical inhibitor of the Wnt/β-catenin signaling pathway that can block the expression of the entire signaling pathway. Therefore, we used both chloral hydrate and Dkk1 treatment to assess the relationship between cilia and the Wnt/β-catenin signaling pathway in MSCs. We subjected four groups, MSCs only (MSC), MSCs co-cultured with HGC27 (MSC+HGC27), DKK1-treated MSCs co-cultured with HGC27 (MSC+HGC27+DKK1) and chloral hydrate-treated MSCs co-cultured with HGC27 (MSC+HGC27+chloral hydrate), to OS induction. We found that treatment with DKK1(10 nM) did not significantly prevent the formation of cilia, with some cells still retaining as many as 3-5 cilia. Furthermore, γ-tubulin was expressed in high density in both the cytoplasm and the nucleus (Figure 6A). As shown in Figure 6B, 6C, Western blot results also showed that DKK1 had no significant inhibitory effect on the increased expression of acetylated α-tubulin and γ-tubulin in the presence of HCG-27 cells. Meanwhile, immunofluorescence results showed that the enrichment of β-catenin in the nucleus was significantly reduced (Figure 6D), and the fluorescence intensity of Wnt3a was also significantly attenuated by the addition of DKK1 (Figure 6E, 6F). Immunoblotting experiments showed that β-catenin protein expression was highly increased, however, Naked1 and Axin1 proteins were greatly decreased in the group of HGC27+MSC compared with MSC group. DKK1(10 nM) rescued these effects of HGC27 in MSCs (Figure 6G, 6H). The addition of chloral hydrate (10mM) blocked the formation of cilia, which in turn inhibited the conduction of the Wnt/ β-catenin signaling pathway (Figure 6A, 6I, 6J). qRT-PCR analysis also showed that the mRNA levels of Wnt3a and β-catenin were increased. However, the mRNA levels of TCF1 and GSK-3β were decreased in the HGC27+MSC group compared with the MSC group, while addition of DKK1 (10 nM) rescued the effects of HGC27 in MSCs (Figure 6K). These results provided evidence that the abnormal overexpression of cilia caused by gastric cancer activates the Wnt/β-catenin signaling pathway in MSCs.

### Dkk1 partially rescues gastric cancer-induced bone loss due to gastric cancer

**Figure 7:**
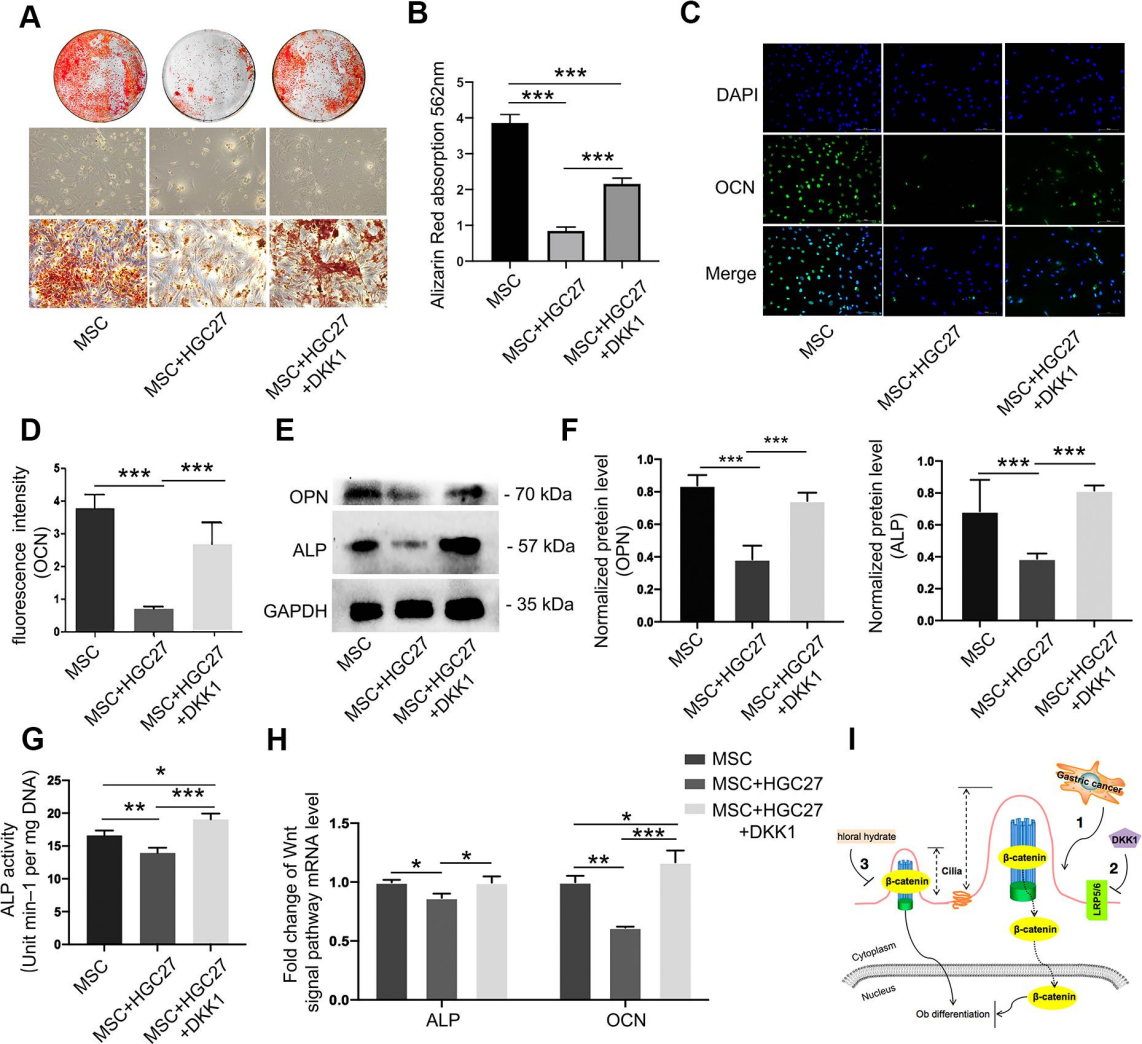
**Dkk1 partially rescued bone loss due to gastric cancer.** (**A**) Bone mineralization levels in the MSC, MSC+HGC27, and MSC+HGC27+DKK1 groups were analyzed by alizarin red staining at day 16 post-OS induction. (**B**) Quantitative mineralization levels based on (**A**). (**C**) Immunofluorescence staining of MSC, MSC+HGC27, and MSC+HGC27+DKK1 to visualize OCN (green) and nuclei (blue). Scale bars, 100 μm. (**D**) Quantitative analysis of the fluorescence intensity in (**C**). (**E**) Western blot analysis of OPN and ALP expression in MSC, MSC+HGC27, and MSC+HGC27+DKK1 groups. (**F**) Quantitative analysis of protein levels in (**E**). (**G**) ALP activity in the MSC, MSC+HGC27, and MSC+HGC27+DKK1 groups at day 3 post-OS induction. (**H**) qRT-PCR results comparing ALP and OCN transcription levels in the MSC, MSC+HGC27, and MSC+HGC27+DKK1 groups on day 3 post-OS induction. Data are shown as mean±SEM. Statistical differences were obtained using One-way ANOVA with post-hoc testing, *, p<0.05, **, p<0.01, ***, p<0.001. NS, not statistically significant, n=3, per-group. (**I**) Schematic diagram of the role of the Wnt/β-catenin pathway under normal and gastric cancer conditions.

We further investigated whether suppression of the Wnt/β-catenin pathway could salvage osteogenic differentiation. Alizarin red staining showed a significant reduction in osteoblast differentiation with little calcium deposition upon co-culture with HGC27, and this effect was partially rescued by DKK1 (Figure 7A, 7B). Immunofluorescence showed that DKK1 rescued the impaired OCN protein expression caused by HGC27 (Figure 7C, 7D). Similarly, DKK1 partially restored osteogenesis in MSCs, as evidenced by restored expression of OPN and ALP (Figure 7E, 7F), ALP activity (Figure 7G), and mRNA levels of ALP and OCN (Figure 7H). These results demonstrated that the osteogenic capacity of MSCs was significantly reduced by abnormal activation of cilia under the influence of gastric cancer cells and that DKK1 restored the osteogenic capacity of some MSCs with little change in cilia length and number. This suggested that gastric cancer cells activated the Wnt/β-catenin signaling pathway through abnormal activation of cilia, thereby attenuating osteoblast differentiation which further led to bone loss.

## DISCUSSION

Once a tumor has metastasized to the bone, it is extremely painful and causes uncontrolled bone remodeling, which is usually incurable [33]. The devastating consequences of bone metastases include pathological fractures, pain, hypercalcemia, and spinal cord and nerve compression syndrome [34]. Understanding the crucial factors of the gastric cancer that influence bone osteogenesis, along with the important proteins and key mechanisms that regulate osteogenic differentiation, is central to the pathophysiology of bone invasion by tumor cells. Surprisingly, based on the clinical samples collected, we found that some patients had slight changes in Ca^2+^, Pi and ALP concentrations and some CT scans showed slight bone cavities before the bone metastasis of the primary gastric cancer had occurred. These data suggested that the gastric cancer might reduce osteoblast differentiation prior to bone metastasis. Therefore, in this study, we investigated the effect of gastric cancer cells on bone tissue by injecting mice with HGC27 gastric cancer cells *in vivo* and by co-culturing osteoblast cells with HGC27 or SGC-7901 *in vitro.* Our results confirmed that osteoblast differentiation was attenuated in the presence of gastric cancer cells. A series of functional tests showed that gastric cancer cells impaired osteoblast proliferation and differentiation, which was associated with an increase in the levels of acetylated α-tubulin and γ-tubulin and with abnormal length and number of cilia. We showed that the abnormal overexpression of cilia caused by gastric cancer cells activated the Wnt/β-catenin signaling pathway in MSCs. Treatment with DKK1 could rescue osteoblast differentiation that was partially inhibited by gastric cancer cells. These findings suggested that gastric cancer regulated osteoblast differentiation and mineralization through the Wnt/β-catenin signaling pathway, which acted downstream of the cilia. This report demonstrates for the first time that gastric cancer directly affects osteoblast differentiation through the cilia/Wnt/β-catenin signaling pathway.

In this study, we used a colony-forming assay and immunofluorescence to show that the proliferation ability of MSCs co-cultured with HGC27 was significantly inhibited compared with untreated MSCs. We also used OS medium to culture MSCs and observe their osteogenic ability. Alizarin red staining, Western blotting, quantitative PCR and immunofluorescence showed that HGC27 and SGC-7901 had a strong inhibitory effect on the osteogenic differentiation of MSCs. We observed that gastric cancer cells could abnormally activate the cilia and regulate osteogenic proliferation and differentiation. Kuehn et al. have reported that the inhibition of cilia is the driving carcinogenic lesion of most clear cell renal cell carcinomas, showing that ciliary body absorption could promote its role in carcinogenesis by stimulating proliferation [35]. Kim et al. showed that when cells lacked Nde1 longer cilia were formed. They showed a delay in the cell cycle from G1 to S, which was rescued in cells lacking cilia [36]. Interestingly, a natural mutation of Nde1 has recently been described in patients with severe microcephaly [37, 38] and defects have also been observed in mice lacking Nde1 [39]. The data of Kim et al. proposed another model in which the microcephaly of patients with Nde1 mutations might be due to the formation of abnormally long cilia, leading to the extended G1 phase and premature cell cycle exit of neuronal progenitor cells [36]. This assumption was consistent with the data of Li et al. [36, 40]. This was sufficient to show that the destruction of cilia stimulates the cell cycle progression [41]. Therefore, inhibition of primary cilia leads to downstream carcinogenic events, but its enhancement results in delayed cell cycle and bone dysplasia. These seemingly contradictory results can be explained by the cell cycle effects of primary cilia. Similarly, for bone tissues affected by gastric cancer, the increase in cilia length led to a prolonged cell cycle, which affected bone formation through a series of actions on bone. However, it is important to note that these *in vivo* and *in vitro* experiments cannot reproduce the complexities of gastric cancer and bone interactions in humans. Gastric cancer does not directly affect bones through primary cilia. The primary cilia of bone cells may interact with certain extracellular proteins., Further research is needed to explore the relationship between gastric cancer, cilia, and bone tissue.

We further found that gastric cancer affected osteoblast differentiation by prolonging the cilia of osteoblasts by abnormally activating the classical Wnt/β-catenin signaling. We unexpectedly found that β-catenin was co-stained with acetylated α-tubulin in femurs of C57BL mice, which demonstrated that β-catenin was located in cilia. Recent studies have also reported cilia regulation of the Wnt/β-catenin signaling pathway [42]. Several members of the Dvl, β-catenin and β-catenin destruction complexes have been shown to be located at the base of the ciliary body, and existing evidence suggests that they can be regulated by components of the TZ protein complex MKS and NPHP modules, which form the eyelashes. The protruding part of the phyllodes regulates the protein entering and leaving the cilia [43]. Many studies have reported on the location of several core components of Wnt in primary cilia [44–48]. Although multiple lines of evidence from gene knockout studies indicate that cilia play a role in regulating Wnt signaling, the literature in this field is still controversial. Regarding the Wnt signaling pathway, we observed that β-catenin accumulated in the cytoplasm and underwent nuclear translocation in MSCs co-cultured with HGC27, ultimately reducing the expression of the osteogenic marker genes including OCN, OPN, and ALP. Moreover, the addition of chloral hydrate blocked cilia formation, which in turn reduced Wnt signaling. The addition of DKK1, an inhibitor of the Wnt/β-catenin signal pathway, had little effect on the expression of cilia, but rescued the osteogenic defects caused by gastric cancer cells. This suggested that cilia might act upstream of the Wnt/β-catenin signaling pathway that regulated osteogenesis. Consistent with this view, Zhou et al. found that sinusoidal electromagnetic fields (SEMFs) increased the peak bone mass of growing rats by promoting osteogenic differentiation/maturation of osteoblasts. This was mediated by Wnt10b of primary cilia that elongated with SEMFs of different intensities, and subsequently activates Wnt/β-catenin signaling [49]. Qiu et al. observed that the expression of Axin2, a direct downstream gene of Wnt/β-catenin signaling in the bones and osteoblasts of conditional Kif3a^OC-CKO^ null mice, was significantly reduced compared to Kif3a^flox/+^ control mice [50]. In addition, Wnt3a induced cytosolic β-catenin accumulation and β-catenin transcriptional activity were significantly reduced in osteoblasts from conditionally Kif3a^OC-CKO^ null mice [50]. However, there are also many studies that have shown that cilia restrict nuclear entry by isolating β-catenin in the ciliary body cavity, thereby inhibiting classical Wnt signaling [51]. The loss of cilia enhances the canonical Wnt response [51]. Thus, despite intense investigation, the exact function of the primary cilia in fine-tuning Wnt signaling remains unclear [52].

Here, we found that in MSCs co-cultured with HGC27 the expression of β-catenin and Wnt3a increased, while the expression of Naked1 and Axin1 as well as the mRNA levels of TCF1 and GSK-3 decreased significantly, which suggested that gastric cancer cells activated the Wnt signaling pathway. Most studies have shown that the Wnt signaling pathway has positive effects on bone tissue. However, we found that the Wnt signaling pathway in bone tissue under the influence of gastric cancer cells negatively regulated osteogenic differentiation. This can be explained by the results of Chen et al. who proved that high levels of Wnt1 and β-catenin expression were associated with advanced, metastatic, hormone refractory prostate carcinoma [53]. De Toni et al. concluded that the Wnt/β-catenin pathway contributed to carcinogenesis and the survival of colorectal tumors by driving the expression of OPG, contributing to cell invasion and metastasis [54]. Abnormal activation of the Wnt/β-catenin pathway has been described in a wide variety of human cancers, such as colon [55], prostate [56] and cutaneous cancer [57], chronic myeloid leukemia [58], and hepatic carcinoma [59]. In addition, some studies have reported that the abnormal activation of the Wnt pathway inhibited osteogenic differentiation. Nemoto et al. suggested that Wnt signaling inhibited cementoblast differentiation and promoted cell proliferation [60]. Jiang et al. suggested that Wnt16 is involved in intramembranous ossification and suppressed osteoblast differentiation through the Wnt/β-catenin pathway [61]. Thus, it was reasonable to assume that the abnormally activated Wnt pathway of gastric cancer also affects the osteoblast Wnt signal pathway, leading to downregulation of OCN, ALP, and OPN protein expression and ultimately leading to inhibition of osteogenic differentiation.

In summary, our data showed that non-bone metastatic gastric cancer may induce bone loss. This effect was mediated by the Wnt/β-catenin pathway, and osteoblast cilia were the upstream effector that controlled this pathway. The accumulation and nuclear translocation of β-catenin and the increased of Wnt3a expression suggested that the Wnt/β-catenin pathway was abnormally activated in the presence of gastric cancers. This activation could significantly inhibit osteogenic differentiation and lead to significant bone loss. Therefore, our study revealed a new mechanism of osteogenic differentiation in which cilia play a key role. This work also suggested that bone loss caused by non-bone metastasis of gastric cancer might be brought about via the cilia/Wnt/β-catenin signaling pathway. This provides new ideas for the prevention of bone loss induced by gastric cancer.

## MATERIALS AND METHODS

### Case collection and inclusion criteria

To investigate whether gastric cancer affects bone tissue, we collected gastric cancer specimens from Chongqing Emergency Medical Center. All clinical trials were conducted in strict compliance with the Helsinki Declaration and relevant regulations of Chinese clinical trials, and approved by the Chongqing Emergency Medical Center Hospital Ethics Committee. Written informed consent was obtained from all participants. Inclusion criteria (patients are required to meet each of the following three conditions): 1) Patients with gastric cancer but without bone metastasis (gastric cancer patients with other, non-bone, metastasis were included in the study); 2) Patients younger than 50 years old (excluding age-related effects on bone mass); 3) Patients without primary bone disease or other diseases affecting bones. In total, we collected 25 specimens that met those criteria. Among these 25 specimens, there were 15 cases of gastric malignant gastric cancer, 5 cases of gastric cardia malignant gastric cancer, 3 cases of malignant gastric cancer of the esophagogastric junction and 2 cases of gastric antrum malignant gastric cancer. Finally, we determined the Ca^2+^, inorganic phosphate, and ALP concentrations in the serum of these 25 patients, and collected patient information that were not within the normal range into Table 1.

**Table 1:** Patients' details.

**Patients**	**Ca2+ (mmol/l)**	**Pi (mmol/l)**	**ALP (U/L)**	**hs-CRP (mg/L)**	**PCT (ug/L)**	**Primary diagnosis name**
Patient1	1.86		26	32		Gastric malignancy
	1.77		31			
	1.98			39.4		
Patient2	1.84	1.95			21.097	Gastric malignancy
Patient3	1.86		242		1.358	Gastric cardia malignant gastric cancer
	1.90		496		1.285
Patient4	2.01	0.46		19.9	1.122	Gastric malignancy
	1.86	0.52		62.9	2.909	
	1.85	0.61	206	91.2		
	1.94	0.33		45.9	3.652	
	1.88	0.42	219	37.2	2.53	
	1.91	0.31	254	72.8	2.73	
	1.72	0.29		77.2	1.62	
Patient5	2.00		31	59.0		Gastric malignancy
	1.98		29			
	1.99		26	25.2		
Patient6	1.77	0.39		135.7	2.559	Gastric malignancy
	1.86	0.56		126.8	3.553	
	1.98	0.51		119.4	3.52	
Patient7	1.86	0.58		188.6		Gastric malignancy
	1.8	0.63		120.7		
	1.98	0.43		195.4		
Patient8	2.00					Gastric malignancy
Patient9		0.76				Gastric malignancy
Patient10			255	39.53	1.01	Gastric malignancy
Patient11	2.02					Gastric malignancy
	2.01					
Patient12	1.80	0.58		43.3		Gastric malignancy
	1.65	0.43		85.3		
	1.81	0.39		127.9		
Patient13	2.01					Gastric malignancy
	1.96					
						
Remark:		:Reduce		:Increase		

### Cell culture and reagents

A murine pre-osteoblastic cell line derived from murine calvaria (MC3T3-E1 clone 4) [62], gastric epithelial cell line GES-1, gastric cancer cell line HGC27 and human gastric cancer cell line SGC-7901 [63] were obtained from the American Type Culture Collection (ATCC). Animal procedures were conducted in accordance with the protocol approved by IACUC of the Chongqing medical University. Sprague-Dawley (SD) rats, 3-4 days old, were immersed in 75% alcohol to death. In a sterile hood, the rat femurs and tibias were dissected free from the surrounding soft tissue. Metaphysis from both ends were resected, and bone marrow cells were collected by flushing the diaphysis with phosphate-buffered saline (PBS) and separated by Histopaque-1083 (Sigma) density gradient centrifugation at 400 g for 20 min. Culturing and differentiation of rat primary osteoblast precursor cells (OPCs) were performed as follows. Primary OPCs were isolated from rat calvarial bone by a serial digestion method. Briefly, calvarial bone was dissected and subjected to sequential digestions in collagenase type I (2mg/ml, EMD, Darmstadt, Germany), Trypsin (0.25%, Corning, Manassas, VA, USA) and collagenase type I (2 mg/ml) for 30 min each. Then the bone chips were subjected to collagenase type I (2 mg/ml) again for 10 min. Cells from this digestion were spun down and plated in α-MEM-containing 10% fetal bovine serum (FBS), 100 U/ml penicillin and 1 mg/ml streptomycin. MC3T3 cells, mesenchymal stem cells (MSCs), and OPC cells were amplified as previously described [64]. For osteoblast differentiation, MC3T3-E1 cells or MSCs were induced with osteogenic medium (OS media:α-MEM (Gibco) containing 10% FBS, 10 mM b-glycerophosphate (Sigma, St Louis, MO, USA), 50 mg/ml ascorbic acid (Sigma) and 10^-8^ M dexamethasone (Sigma) [65]).

### Mouse xenograft studies

HGC27 cells (1×10^8^/mL) were suspended in PBS, sealed, and placed in ice water for later use. Twelve 4-6 weeks old C57BL mice (both male or female) were purchased from the Experimental Animal Center of Chongqing Medical University). Mice were injected with 0.1 mL of HGC27 cell suspension into the ventral side to induced gastric cancer formation and reared in separate cages for 90 days post-injection [66].

### Immunofluorescence

To visualize cilial structures, immunofluorescence was performed using an anti-acetylatedα-tubulin antibody (1:1000, T6793, Sigma) and an anti-γ-tubulin antibody (1:1000, T3320, Sigma) [67]. Briefly, cells were washed with PBS and fixed with 4% paraformaldehyde at room temperature. Fixed cells were permeabilized with 0.05% Triton X-100 and then incubated with the primary antibodies overnight at 4° C. Alexa Fluor 568-conjugated anti-rabbit (1:1000, A-11011, Invitrogen) or Alexa Fluor 647-conjugated anti-mouse (1:1000, A-21235, Invitrogen) antibodies were used as secondary antibody. Counter staining of nuclei was done with DAPI (Sigma) [68]. The experiments were conducted in quadruplicate. The same treatment was used for Ki67 (1:1000, J3009, Santa Cruz Biotechnology, USA), PCNA (1:1000, A5324, Selleckchem, USA), β-catenin (1:200, A5038, Bimake, USA) staining and Wnt3a (1:200, 2721, Cell Signaling Technology, USA) staining as well as for Osteocalcin (OCN) (1:1000, 16157-1-AP, Proteintech, USA) staining. Ki67 was visualized using a FITC-conjugated goat anti-rabbit (1:1000, 0110119-0100, BBI) antibody.

### Colony-forming assay

For the colony-forming assay, 1000 MSCs in 2 mL of medium were added to each well of a 6-well plate and cultured for 10 days. Subsequently, the cells of the treatment group were co-cultured with HGC27 cells. After culturing the cells under standard conditions for 14-18 days, the medium was removed and the cells were washed 3 times with PBS. Next, 4% formaldehyde was added at 1 mL/well for 15 minutes to fix the cells. After discarding the fixation solution, the cells were stained with crystal violet dye, and the cells were photographed and counted.

### Quantitative PCR

Total RNA was extracted from cultured MSCs with Trizol reagent (Invitrogen, Carlsbad, CA) following the manufacturer’s instructions. cDNA was synthesized from 3 mg total RNA by RNA to cDNA EcoDry Premix kit (Clontech, Palo Alto, CA, USA). qPCR was performed with SYBR Green PCR master Mix (Invitrogen). Primers were designed with a primer design tool (Integrated DNA Technologies, Beijing, China). All reactions were run in triplicate and normalized to GAPDH expression. Calculations were performed according to the 2^-ΔΔCT^ method [69]. The sequence of each primer pair is shown in Table 2.

**Table 2:** The sequences of PCR primers.

**Gene**	**Sequence(5'-3')**
ALP	Sense: 5'-GCACCTGCCTTACCAACTCT-3' Antisense: 5'-GGACCTGAGCGTTGGTGTTA-3'
Osteocalcin	Sense: 5'-TTCTGCTCACTCTGCTGACC-3' Antisense: 5'-GGCGGTCTTCAAGCCATACT-3'
Wnt3a	Sense: 5'-AGCGAGGACATCGAGTTTGG-3' Antisense: 5'-CTTCTCCACCACCATCTCCG-3'
GSK-3β	Sense: 5'-AACTCCACCAGAGGCAATCG-3' Antisense: 5'-AAGCGGCGTTATTGGTCTGT-3'
β-catenin	Sense: 5'-ATCATTCTGGCCAGTGGTGG-3' Antisense: 5'-GACAGCACCTTCAGCACTCT-3'
TCF-1	Sense: 5'-AGGAGGCGAAGAAGCCAATC-3' Antisense: 5'-GATAATGCATGCCACCTGCG-3'
GAPDH	Sense: 5'-GACCACAGTCCATGCCATCA-3' Antisense: 5'-GTCAAAGGTGGAGGAGTGGG-3'

### Histology staining

Mice tibiae were excised, fixed for 24 h in 10% natural buffered formalin, and decalcified in 10% EDTA for 1–2 weeks at 4 C. The samples were embedded in paraffin, sectioned for 5 μm and stained with H&E.

### Immunohistochemistry staining (IHC)

Deparaffinized slides were stained using antibodies against osteopontin (1:200, 225952-1-AP, Proteintech) and p-β-catenin (1:200, BS5057, Bioworld Technology, Inc.) by immunohistochemistry (with three replicates each). Retrieved tissues were fixed, decalcified in 10% formalin and embedded in paraffin 24 h post-treatment. After being washed with PBS, tissues were incubated with biotin-labeled secondary antibody for 30 min, followed by incubation with streptavidin-HRP conjugate for 20 min at RT. The presence of the expected protein was visualized by DAB staining and examined under a microscope. Stains with control IgG were used as negative controls.

### Bone micro-CT analysis

A quantitative analysis of the gross bone morphology and microarchitecture was performed with the Micro-CT system (Skyscan1172) and analyzed using CTAn software (Skyscan). Femurs from C57BL mice with or without HGC27 treatment were fixed, scanned, and reconstituted as three-dimensional images. Cancellous bones were evaluated in the distal femur metaphysis. About 125 slices (1.5 mm) of bone were measured to determine BV/TV (%), Tb.N (mm/1), Tb.Th (mm) and trabecular spacing (Tb.Sp, mm).

### Alizarin red S staining

Mineralized matrix nodules were stained for calcium precipitation by means of Alizarin Red S staining as described in [70]. Briefly, the slides were stained with 40 mmol/L of Alizarin red solution (pH 4.4) for 40 min at room temperature and rinsed twice with deionized water. The images of stained cells were captured using a phase contrast microscope with a digital camera (IM50, Leica, Germany). Slides were then destained using 10% cetylpyridinium chloride in 10 mM sodium phosphate (pH 7.0) and quantified by measuring the optical density at 562 nm. The experiment was done in triplicate.

### Western blot

Cells were lysed with NP40 buffer (1% NP-40, 0.15 M NaCl, 50 mM Tris, pH 8.0) containing protease inhibitors (Sigma). Protein concentration was measured using the bicinchoninic acid (BCA) protein assay reagent (Pierce, Rochford, IL, USA). Equal amounts of total protein (about 20 μg) were denatured in SDS and separated in 10% SDS-PAGE gels. Proteins were transferred to polyvinylidene difluoride membranes in buffer containing 25 mM Tris, 192 mM glycine and 20% methanol. The membranes were blocked with 5% skim milk, incubated with primary antibody overnight at 4° C, and then incubated with horseradish peroxidase (HRP)-conjugated goat anti-rabbit IgG antibody (1:10,000, A-11034, Novex, CA, USA) or horseradish peroxidase (HRP)-conjugated goat anti-mouse IgG antibody (1:5,000, #7074, Cell Signaling technology) at room temperature for 1 h. Visualization was performed with Western Bright ECL HRP (Advansta). Primary antibodies used were: OPN (1:1,000, 22952-1-AP, Proteintech), ALP (1:1,000, 18507-1-AP, Proteintech), β-catenin (1:1,000, 51067-1-AP, Proteintech) Wnt3a (1:1,000, C64F2, Cell Signaling), Axin1 (1:1,000, C76H11, Cell Signaling), Naked1 (1:1,000, C30F10, Cell Signaling). Anti-GAPDH (1:1,000, YT5052, ImmunoWay) was used as the internal control [71].

### Alkaline phosphatase enzyme activity

Cells were washed with PBS and lysed with lysis buffer (0.2% NP-40, 1 mmol/L MgCl_2_). Lysates were centrifuged and enzyme activity was assayed in the supernatant by the addition of 10 mmol/L of p-nitrophenyl phosphate as a substrate in 0.1 mol/L glycine buffer, pH 10.4, containing 1 mmol/L ZnCl_2_ and 1 mmol/L MgCl_2_, followed by incubation at 30° C. The quantity of p-nitrophenol product formed was recorded by monitoring the absorbance at 405 nm at regular time intervals. Protein content was determined using the BCA protein assay kit (Sigma, UK). The specific activity of alkaline phosphatase (ALP) was expressed as nmol/min per milligram of protein.

### Statistical analyses

All data are presented as mean ± standard error of the mean (SEM) (n=3 or more). Statistical analysis was performed using SPSS-17.0 software. Data were analyzed using one-way analysis of variance (ANOVA), and Tukey’s HSD test was applied as a post-hoc test if statistical significance was determined. Statistical significance for the two groups was assessed using Student’s t-test. Differences were considered significant if P<0.05.

## Supplementary Material

Supplementary Figure 1
