# Supplementary material for: Primary cilia regulate gastric cancer-induced bone loss via cilia/Wnt/β-catenin signaling pathway
Source: Aging (Albany NY). 2021 Mar 9;13(6):8989–9010. doi: 10.18632/aging.202734 (PMC8034975; doi:10.18632/aging.202734)
Supplement: Supplementary Figure 1 [file aging-13-202734-s001.pdf]

SUPPLEMENTARY FIGURE

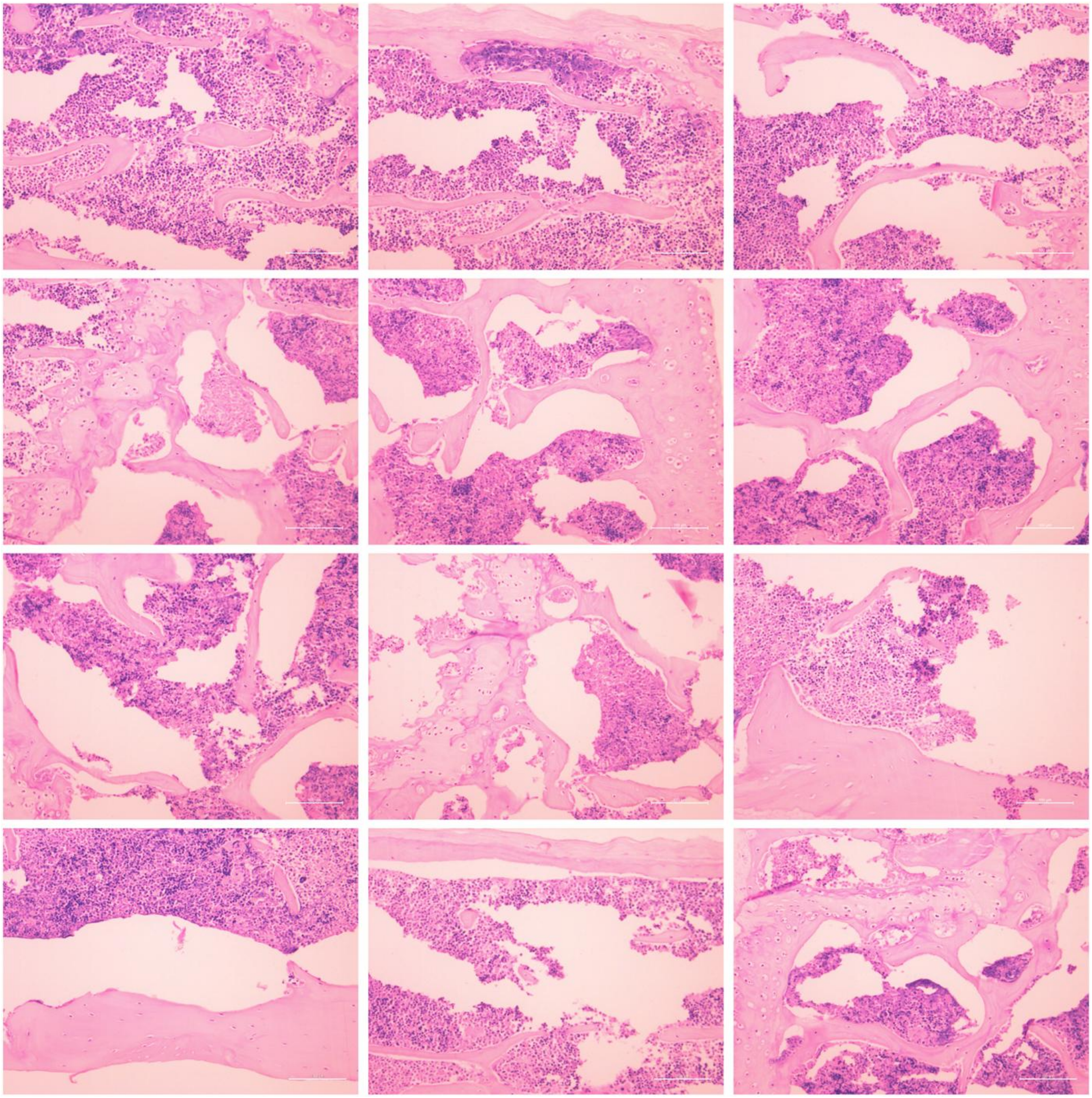

Supplementary Figure 1. H&E staining of tumor-forming bone tissue sections of nude mice on 90 days.
